# Efficacy of Lidocaine Infusion in the Management of Chronic Myofascial Pain and Intractable Migrainous Headache in a Patient With Hypermobile Ehlers-Danlos Syndrome: A Case Report

**DOI:** 10.7759/cureus.103015

**Published:** 2026-02-05

**Authors:** Roy Sebastian, Thelma Wright, Seung J Lee, Kanchana Gattu

**Affiliations:** 1 Anesthesiology, University of Maryland Medical Center, Baltimore, USA; 2 Anesthesiology, University of Maryland School of Medicine, Baltimore, USA

**Keywords:** dysautonomia, hypermobile ehler-danlos syndrome, lidocaine infusion, postural orthostatic tachycardia syndrome (pots), trigger point injection

## Abstract

Hypermobile Ehlers-Danlos Syndrome (hEDS) is the most common type of inherited connective tissue disorder, often presenting with chronic widespread myofascial pain, autonomic dysfunction, soft-tissue fragility, and psychiatric comorbidities. Pain is often multifactorial and refractory to conventional therapies. We describe a young adult with longstanding hypermobility, recurrent subluxations, chronic periscapular pain, migrainous headaches, and dysautonomia who achieved marked clinical improvement with lidocaine-based therapies, including trigger-point injections (TPIs) and scheduled intravenous lidocaine infusion. TPIs provided more than 50% relief of the myofascial pain for over one month, which is significantly longer than the typical duration of conservative treatments. A lidocaine infusion was subsequently administered to provide extended and more widespread pain relief, resulting in a favorable clinical outcome. This case illustrates the value of lidocaine as part of a multimodal strategy in managing complex hypermobility-related pain syndromes.

## Introduction

The Ehlers-Danlos syndromes (EDS) comprise a clinically and genetically heterogeneous group of heritable connective tissue disorders (HCTDs) characterized by joint hypermobility, skin hyperextensibility, and tissue fragility. The Villefranche Nosology, published in 1998, delineated six EDS subtypes and served for many years as the standard framework for clinical diagnosis [1]. Since its introduction, numerous additional EDS subtypes have been identified, along with pathogenic variants in several previously unrecognized genes. In response to these advances, the International EDS Consortium revised the classification system to recognize 13 distinct EDS subtypes, most of which are associated with mutations in collagen-encoding genes or genes involved in collagen modification [2]. The 2017 diagnostic criteria for hypermobile Ehlers-Danlos syndrome (hEDS) require the presence of generalized joint hypermobility (as assessed by an age-adjusted Beighton score), fulfillment of at least two systemic or historical criteria, and exclusion of other heritable or acquired connective tissue disorders. Individuals who exhibit joint hypermobility and chronic pain but do not meet the full diagnostic criteria for hEDS are classified as having a hypermobility spectrum disorder (HSD). While both conditions exist along the same clinical spectrum, hEDS represents a more specific diagnosis, whereas HSD encompasses a broader population lacking the systemic and familial features characteristic of hEDS [2]. Clinical manifestations include joint hypermobility, chronic musculoskeletal pain, joint instability, fatigue, gastrointestinal symptoms, headaches, autonomic dysfunction, immune-related abnormalities, pelvic organ prolapse, and soft tissue weakness, all of which contribute to increased tissue fragility and functional disability [3]. Despite its relatively high prevalence of approximately one in 500 individuals, the underlying pathophysiology of hEDS remains poorly understood [3]. As a result, affected individuals frequently experience delayed, missed, or incorrect diagnoses, often following prolonged periods of significant morbidity and acute symptom exacerbations.

On a structural level, the dysfunction of collagen in hEDS causes loose connective tissue, leading to a lack of tensile strength with joint instability. In an attempt to counter the lack of passive support, a reliance on musculature is necessary for the preservation of joint stability, giving rise to overuse compensation, particularly in the muscles, leading to eventual exhaustion [4]. This state of repetitive use encourages micro-injury within the musculature, presenting as microscopic tears within the muscles [5]. Over the course of time, these areas of injury can progress to the development of myofascial trigger points (MTrPs), which are hyperirritable spots within bands of taut muscles that provoke deep aching pain with characteristic referral pain phenomena. Posture dysfunction, a common component within patients with hEDS, further increases strain on the musculature, contributing to MTrPs being difficult to eradicate, resulting in irremissible chronic pain syndromes [5].

In addition to peripherally acting musculoskeletal factors, there is a growing body of evidence that central sensitization is a contributing mechanism to the pain experienced in hEDS. Central sensitization has been identified in adolescents as well as adults, supporting the hypothesis that it is an integral part of the pathophysiology of nociplastic pain in patients with hEDS [6]. Central sensitization can be identified when there is a loss of endogenous pain inhibitory control after exercise [6].

There is growing evidence that patients with hEDS have an increased incidence of immune dysfunction and mast cell activation disorder compared with the normal population, which also adds to the complexities of treatment [7]. The patient with hEDS suffering from chronic myofascial pain is typically non-responsive to the usual conservative management. Lidocaine, when administered locally through trigger-point injections or intravenously, has been described as a useful alternative treatment for patients with myofascial and neuropathic pain who do not respond to conservative therapies. Its therapeutic effects are attributed to stabilization of neuronal membrane potential, interruption of abnormal pain pathways, and modulation of inflammatory responses. Specifically, lidocaine has been shown to decrease proinflammatory cytokines, including interleukin-1 (IL-1), interleukin-6 (IL-6), interferon-γ, and tumor necrosis factor-α (TNF-α), while increasing anti-inflammatory cytokine IL-10 [8].

We report the case of a patient with hEDS presenting with generalized myofascial pain and daily migrainous headaches, who responded significantly to a treatment plan involving lidocaine, and explore the implications of this phenomenon for our current understanding of hEDS, a condition characterized by musculoskeletal problems and daily headaches.

## Case presentation

A young adult female in her early twenties with a medical history significant for hypermobility, joint pains involving multiple joints, including neck, shoulders, wrists, fingers, hips, knees and ankles, chronic migrainous headache, gastroesophageal reflux disease, possible colon prolapse, bladder prolapse, recurrent urinary tract infections (UTIs), postural orthostatic tachycardia syndrome (POTS), easy bruising, attention-deficit/hyperactivity disorder (ADHD), sleep disturbance, and post-traumatic stress disorder (PTSD). She described recurrent subluxations affecting the fingers, shoulders, hips, knees, and ankles, with only partial symptomatic pain relief from nonsteroidal anti-inflammatory drugs (NSAIDs) and acetaminophen. Upon presentation to the pain clinic, her chief complaints were chronic right periscapular pain and daily migrainous headache that had been progressively worsening over the past year. The symptoms were exacerbated by physical activities and improved minimally by heat, ice, transcutaneous electrical nerve stimulation (TENS), and analgesics. She was maintained on Lisdexamfetamine dimesylate with regular psychotherapy and psychiatric follow-up.

**Figure 1:**
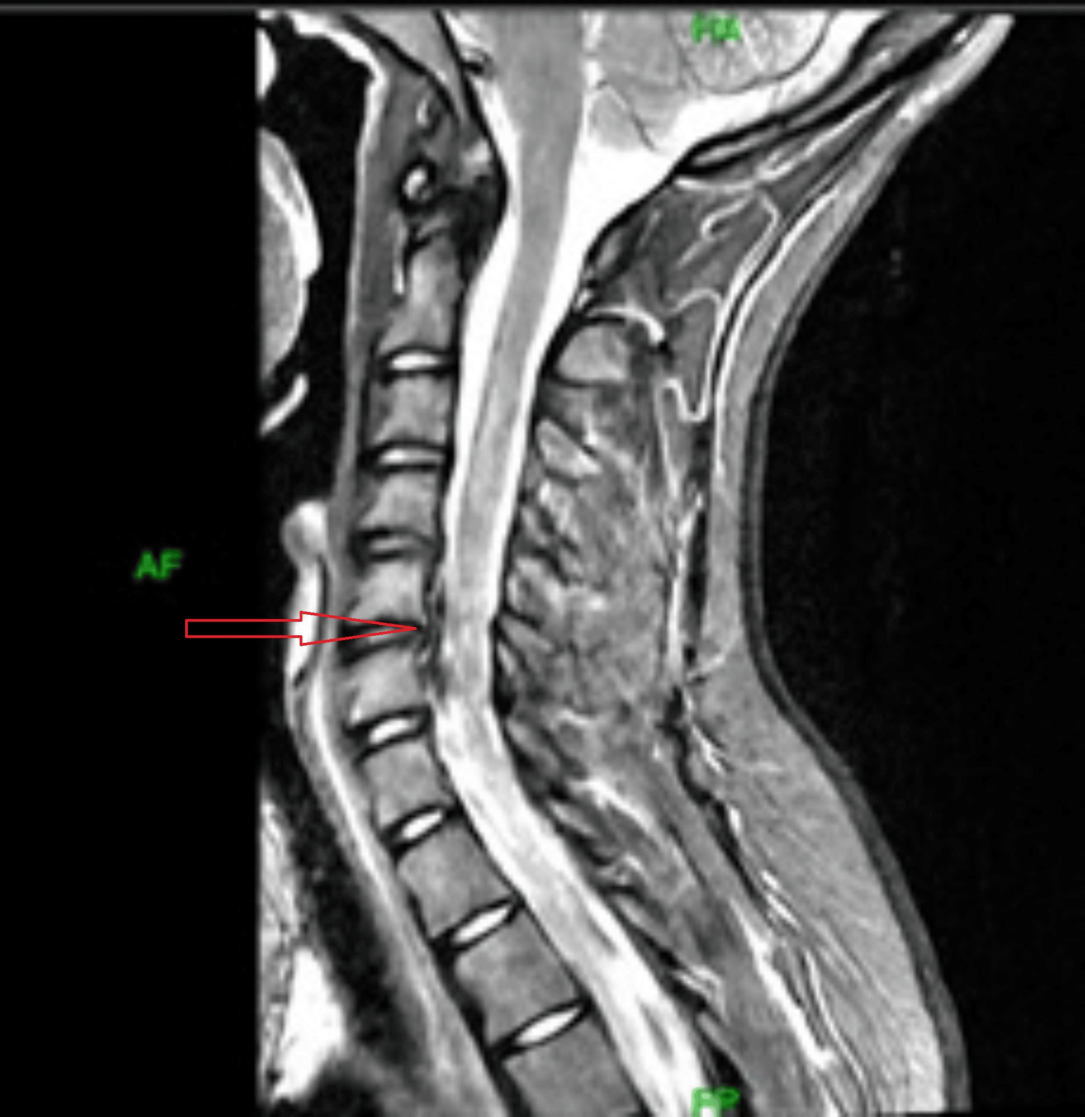
MRI of cervical spine: sagittal view. MRI of the cervical spine shows minimal degenerative changes - presence of osteophytes at the C5 and C6 posterior vertebral endplates indicated by the red arrow, without significant foraminal or spinal canal stenosis.

**Figure 2:**
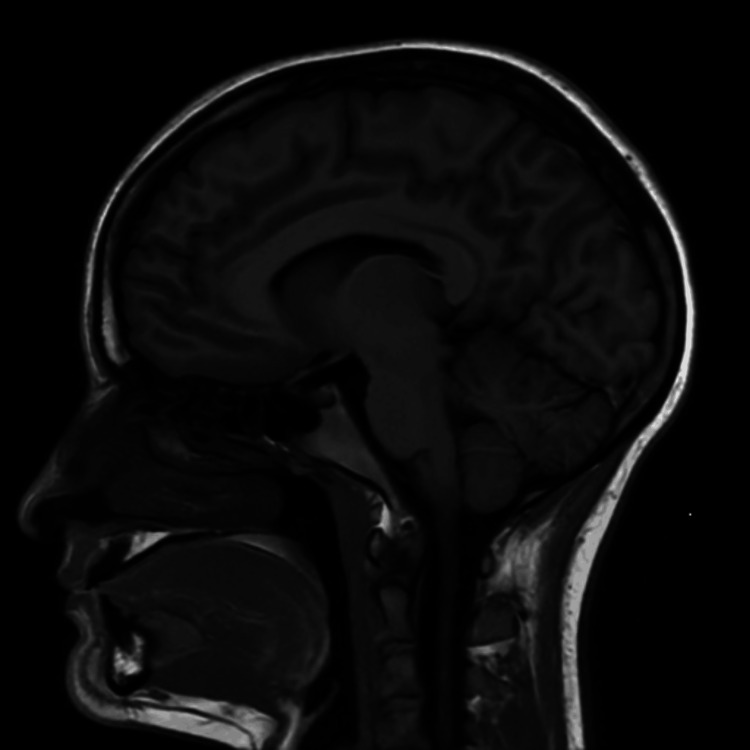
MRI of the brain without contrast showing normal cerebellar tonsils without ectopia or bulging through the foramen magnum.

Physical examination with the referring provider revealed a Beighton score of 5/9 [9], full range of motion in all major joints, positive thumb sign, bilateral piezogenic papules, and, in general, soft hyperextensible skin; neurologic examination remained unremarkable. Diagnostic studies showed minimal cervical degenerative changes, which include osteophytes over the posterior vertebral end plates of C5 (inferior end plate) and C6 (superior end plate) without stenosis on cervical magnetic resonance imaging (MRI) (Figure 1) and an unremarkable brain MRI (Figure 2), with intermittent tachycardia in cardiac monitoring.

Genetic testing using a connective tissue gene panel identified a heterozygous variant of uncertain significance in the TGFB2 gene (c.185A>C, p.Glu62Ala). While TGFB2 is associated with other connective tissue disorders, such as Loeys-Dietz syndrome, it has not been definitively linked to hEDS, which currently has no confirmed single-gene cause. Therefore, this variant does not confirm or explain the patient’s hEDS diagnosis and does not alter clinical management. It is reported for completeness and may be reclassified in the future as additional evidence becomes available.

During her initial pain clinic evaluation, she described her right periscapular pain as 8/10 in the visual analog scale (VAS) with intermittent throbbing quality and a functional pain score of 5/10 [10]. The headaches were characterized by throbbing, aching pain localized to the occipital region. They were not associated with photophobia, phonophobia, nausea, or vomiting and were exacerbated by neck movement. The patient’s headache characteristics and history did not meet the International Classification of Headache Disorders (ICHD) criteria for migraine with aura, medication-overuse headache, cluster headache, or idiopathic intracranial hypertension. The patient reported no relief from over-the-counter analgesics used for headache management. She reported being functionally independent. Her physical examination findings were unchanged from those documented by the referring provider, including myofascial trigger points over the right periscapular area. Myofascial trigger points are discrete hyperirritable spots within a taut band of skeletal muscle that are tender on palpation and may reproduce the patient’s typical local or referred pain. Clinically, they present as focal areas of deep, aching muscle pain that are aggravated by pressure or use of the involved muscle and are a common physical finding in myofascial pain syndromes [11]. A multimodal treatment approach was initiated, including physical therapy, behavioral health support, and consideration of procedural intervention. During her second clinic visit in early September 2025, she underwent a right periscapular trigger point injection (TPI) at five separate sites, each receiving 0.5 mL of 1% lidocaine, followed by dry needling. This intervention provided substantial symptomatic relief, lasting over one month and resulting in more than a 50% improvement in both pain and function.

Subsequently, after a transient pain flare following overexertion, the patient required treatment in the emergency department (ED) and brief administration of ketorolac and oxycodone. Her symptoms stabilized with the addition of low-dose as-needed metaxalone to the short course of oxycodone. At follow-up in the pain clinic after the ED visit, the patient continued to experience generalized joint pain and focal periscapular pain. This prompted a second set of trigger-point injections to the periscapular region in mid-October 2025, which again provided significant relief of the focal pain and resulted in sustained functional improvement. Given the chronicity of the patient’s widespread pain, the pattern of diffuse myofascial hyperalgesia, daily intractable migrainous headaches, and a favorable response to prior local anesthetic injections, a trial of intravenous lidocaine was initiated to address suspected central sensitization and diffuse myofascial pain. In late October 2025, the patient received a 300-mg intravenous lidocaine infusion, prepared from 2% lidocaine diluted in 250 mL of 0.9% sodium chloride and administered at an infusion rate of 500 mL/hour. The patient did not experience any adverse effects during or after the lidocaine infusion.

She described a significant decrease in generalized pain after the initial lidocaine infusion, noting that her VAS score had decreased to 6/10, her functional pain scale had improved to 3/10, and her headache days had been reduced by 33%. After the second infusion, which was two weeks later, her VAS score improved to 4/10, the functional pain scale was reduced to 2/10, and headache frequency decreased by 50%. This positive clinical response prompted scheduling her for two more months of monthly lidocaine infusions, to be reconsidered at subsequent visits if pain scores began to worsen or headache frequency increased again. Following the fourth infusion, the patient’s average pain level stabilized at 2/10, with a functional pain score of 1/10 and fewer than seven headache days per month. Significant relief in headache intensity was achieved with acetaminophen. No further lidocaine infusions were planned, and the improvement in pain symptoms has remained stable. A shared decision was made, following discussion with the patient and her significant other, to repeat lidocaine infusions if her generalized pain intensity worsens or her headache frequency increases.

## Discussion

Hypermobile EDS (hEDS) is a uniquely challenging clinical entity, as it stands in contrast to most other EDS subtypes, which have identifiable pathogenic variants in genes of collagen and/or extracellular matrix-related genes [12]. Current diagnostic criteria are strictly clinical, since hitherto proposed candidate genes failed to show reproducible pathogenic variants in well-characterized patient cohorts. Recent genetic research has started to shed light on possible mechanisms of the disease: a genome-wide association meta-analysis involving 1,815 cases of hEDS and 5,008 controls identified two genome-wide significant loci, including a regulatory region close to ACKR3, a gene involved in neuroimmune and pain-signaling pathways [12]. Gene-based and transcriptomic analyses from the same cohort highlighted variants associated with SLC39A13 - a zinc transporter previously associated with spondylodysplastic EDS - and PSMC3, a gene implicated in the development of the central nervous system. All together, these results support a polygenic model where, in hEDS, the combined effects disturb connective tissue integrity, remodeling of the extracellular matrix, and neural-stromal signaling [5]. Until these associations are validated across independent populations, robust molecular diagnostic markers are, nevertheless, not available, and diagnosis still must refer to clinical criteria.

Chronic pain and headache are highly prevalent in hEDS, affecting up to 90% and 70% of patients, respectively [13, 14]. Symptoms result from multiple converging pathophysiological processes: connective tissue fragility promotes joint instability, recurrent subluxations, and microtrauma, producing persistent nociceptive input; abnormal collagen composition and impaired proprioception foster dysfunctional muscle recruitment, leading to widespread myofascial pain; and central sensitization, a hallmark of chronic pain in hEDS, lowers nociceptive thresholds and amplifies mechanical and thermal hypersensitivity [15]. Impaired regulation of the autonomic nervous system, particularly postural orthostatic tachycardia syndrome, is an additional headache burden due to impaired cerebrovascular regulation [16]. This self-reinforcing pain phenotype often requires a multimodal approach integrating physical therapy, psychological support, and targeted interventions for peripheral and central pain generators.

Within this multimodal framework, intravenous lidocaine represents an attractive therapeutic option, given its numerous analgesic mechanisms. Lidocaine inhibits voltage-gated sodium channels, suppressing ectopic discharges from sensitized peripheral nerves and reducing ongoing nociceptive signaling. It also modulates central pain pathways by attenuating dorsal horn hyperexcitability and reducing central sensitization through effects on N-methyl-D-aspartate (NMDA) receptors and calcium channels [17]. Additional anti-inflammatory properties, including suppression of neuroinflammatory cytokines, may also explain why lidocaine provides relief that outlasts its short plasma half-life [18]. In refractory headache disorders such as chronic migraine and status migrainosus, intravenous lidocaine has been shown to clinically benefit in reducing headache severity and frequency, especially as a transitional therapy when standard treatments fail [19].

Lidocaine is primarily metabolized in the liver to monoethylglycinexylidide and glycinexylidide and is subsequently excreted by the kidneys; therefore, hepatic or renal dysfunction increases the risk of toxicity. The drug demonstrates non-linear pharmacokinetics, with a half-life of approximately 100 minutes for infusions lasting less than 12 hours and up to 190 minutes for infusions exceeding 12 hours [20]. Continuous infusion beyond 24 hours or use in patients weighing less than 40 kg is not recommended, and clinical effects typically resolve within two to six hours after discontinuation [21]. Lidocaine has a narrow therapeutic window, such that small increases in plasma concentration may result in toxicity. Intravenous dosing typically ranges from 5 to 7.5 mg/kg, with a maximum total dose of 500 mg, administered at an infusion rate of 500 mL/hour [22]. Toxic plasma concentrations are typically observed at 9-10 mg/mL, with common adverse effects including drowsiness, light-headedness, perioral numbness, tinnitus, nausea, and bradycardia, and severe toxicity manifesting as seizures, cardiac arrhythmias, coma, or cardiopulmonary arrest, depending on the total dose and rate and duration of infusion.

Despite the need for monitored administration due to risks such as cardiac conduction abnormalities and neurocognitive effects [23], IV lidocaine remains a valuable therapeutic adjunct for patients with hEDS, especially those with features of myofascial hyperalgesia, autonomic dysfunction, and central sensitization, illustrated in this case. 

## Conclusions

This particular case illustration clearly supports the effectiveness of lidocaine treatments, such as Lidocaine injection and intravenous Lidocaine infusion, in giving significant symptomatic relief to patients with hypermobile Ehlers-Danlos syndrome suffering from myofascial pain and migrainous headaches, which are particularly recalcitrant. The analgesic and anti-inflammatory effects of Lidocaine make it particularly useful in treating myofascial pain syndromes, which have a peripheral origin, apart from the central sensitization mechanisms that are implicated in the pathophysiology of headaches, making it theoretically useful in treating a particular variety of headaches. This particular case clearly supports the need to conduct more research regarding Lidocaine treatment in patients with hypermobility-related headaches. The clinical evidence base for lidocaine in chronic myofascial and related pain conditions related to hEDS remains limited, consisting largely of small randomized controlled trials, observational studies, and case reports with heterogeneous protocols and short follow‑up. Existing trials suggest that systemic or trigger‑point lidocaine can provide short‑term analgesia in selected patients, but effects beyond the immediate post‑infusion or short follow‑up period are inconsistent, and robust data on long‑term efficacy, optimal dosing regimens, and patient selection are still lacking.
